# Sensitivity, Specificity and Accuracy of Androgen Deficiency in Ageing Male (ADAM) Questionnaire for the Clinical Detection of Androgen Deficiency in the Male Population in Pakistan

**DOI:** 10.7759/cureus.11788

**Published:** 2020-11-30

**Authors:** Sidra Naz, Nikeeta Mandhan, Prem Shankar, Kuldeep Raj, Sidra Memon

**Affiliations:** 1 Internal Medicine, University of Health Sciences, Lahore, PAK; 2 Internal Medicine, Dr. Ruth K. M. Pfau Civil Hospital, Karachi, PAK; 3 Internal Medicine, Dow University of Health Sciences, Karachi, PAK; 4 Internal Medicine, Jinnah Sindh Medical University, Karachi, PAK

**Keywords:** testosterone deficiency, pakistan, adam questionnaire

## Abstract

Introduction: Androgen deficiency in relation to the increasing age is quite prevalent worldwide. However, diagnosing it in low-income countries is quite a challenge due to cost concerns. Through this study, we plan to measure the sensitivity and specificity of the Androgen Deficiency in Ageing Male (ADAM) questionnaire in the Pakistani population.

Methods: A cross-sectional survey study was conducted from September 2019 to November 2019 in a Pakistani tertiary care hospital. Two hundred and fifty-five participants belonging to ages 30-69 years completed the ADAM Questionnaire in the out-patient department. Venous blood samples were taken to check serum total testosterone levels.

Results: The ADAM questionnaire revealed 90.12% sensitivity, 41.3% specificity, 45.34% positive predictive value, 90.80% negative predictive value, and 61.29% accuracy in the Pakistani population.

Conclusion: Low specificity and positive predictive value have been shown by the ADAM questionnaire. Hence, it cannot be used as a diagnostic tool to detect androgen deficiency, replacing the blood sample.

## Introduction

Age-related androgen deficiency (AD) is a well-documented phenomenon in a range of studies. As a part of the normal ageing process, levels of testosterone fall around 0.5 to 2% per year starting from the fifth decade of life [1]. Deficiency of testosterone leads to a variety of symptoms including sexual dysfunction, psychological problems like depression and cognitive disability and physical symptoms like fatigue, osteoporosis and loss of body mass [2]. Testosterone deficiency can also predispose ageing males to atherosclerosis, leading to a number of cardiovascular diseases [3].

Serum testosterone level is the best confirmatory test to diagnose AD. Testosterone assays are not easily accessible in resource-poor countries like Pakistan, even though it is the gold standard. Henceforth, consistent efforts have been made to formulate questionnaires for screening of patients with AD [4-5]. Many questionnaires have been developed for screening patients with suspected androgen deficiency, however the most widely used questionnaire is the Saint Louis University Androgen Deficiency in Ageing Male (ADAM) questionnaire. It has three items: energy, mood and sexual function [4].

There is truly little research conducted on screening and diagnosis of androgen deficiency in Pakistan. The goal of this study is to measure the sensitivity and specificity of the Androgen Deficiency in Ageing Male (ADAM) questionnaire in the Pakistani population. It may help clinicians choose a screening tool for testosterone deficiency.

## Materials and methods

A cross-sectional survey was conducted from September 2019 to November 2019 in the Outpatient Department (OPD) of internal medicine in a tertiary care hospital in Pakistan. The study included 255 male participants age 30-69 years. These participants were suspected of testosterone deficiency based on their history and symptoms. Participants with recent illnesses, comorbidities such as renal disease, chronic liver disease and malignancies were excluded from the study, as these diseases may affect the measurement of total testosterone. Those who were on androgen therapy were also excluded.

The 10-item ADAM questionnaire was completed by the participants. The questions were asked by researchers themselves and each question was explained to participants. If the participants chose Yes to either one of the questions: decrease in libido or decrease in strength of erection or any other three questions, their ADAM test was positive [5]. Venous blood samples were collected for total testosterone levels. Liquid chromatography was used to measure total testosterone. To avoid any variation, the staples were collected between 10 am to 12pm. As per American Urological Association, AD is defined as total testosterone less than 8mmol/l, which was our basis for the diagnosis [6].

Statistical Package for Social Sciences® software version 23.0 (SPSS; IBM Corp., Armonk, NY, USA) was used for data analysis. For numerical variables, data were expressed as means ± standard deviations. Frequencies and percentages were used for categorical variables. Using analysis of variance (ANOVA), serum testosterone levels were checked for different age groups. Chi-square test was used to compare the ADAM test for different age groups. An online calculator (MedCalc, Ostend, Belgium) was used to calculate the sensitivity, specificity, positive predictive value, and negative predictive value for the overall ADAM questionnaire. A p-value less than 0.05 indicated that there is a difference in ADAM test and serum testosterone levels.

## Results

The mean age of participants of 51 ± 13 years. A total of 168 participants had a positive ADAM test. The positive rate for the ADAM questionnaire increased significantly as the age increased (Table 1). The mean testosterone level 17.2 ± 7.1 nmol/l. The mean testosterone level significantly decreased with age (Table 2).

**Table 1:** Age versus ADAM Questionnaire Result * means significant result

Age	Androgen Deficiency in Ageing Male (ADAM) questionnaire		P-value
	Positive, n (%)	Negative, n (%)	
30-39 (n=13)	3 (23.0%)	10 (77.0%)	0.0002*
40-49 (n=46)	25 (54.3%)	21 (45.6%)	
50-59 (n=91)	59 (64.8%)	32 (35.2%)	
60-69 (n=105)	81 (77.1%)	24 (22.85%)	

**Table 2:** Mean Total Testosterone Level in Different Age Groups * means significant result

Age	Total Testosterone nmol/l (mean ± standard deviation)	P-value
30-39 (n=13)	20.2 ± 7.2	0.001*
40-49 (n=46)	18.7 ± 7.7	
50-59 (n=91)	14.9 ± 6.9	
60-69 (n=105)	10.3 ± 6.2	

Our results indicated 90.12% sensitivity, 47.31 % specificity, 45.34% positive predictive value (PPV), 90.80% negative predictive value (NPV) and 61.29% accuracy of the ADAM questionnaire (Table 3).

**Table 3:** Sensitivity and Specificity of the ADAM questionnaire

Androgen Deficiency in Ageing Male (ADAM) questionnaire	Total Testosterone		Sensitivity	Specificity	Positive Predictive Vale	Negative Predictive Value	Accuracy
	Low	Normal	90.12%	47.31%	45.34%	90.80%	61.29%
Positive	73	88					
Negative	8	79					

## Discussion

Testosterone deficiency is common, such that 6% of adult males suffer from testosterone deficiency [7]. The incidence increases with increasing age and illness [7]. A meta-analysis concluded that decreased testosterone levels can lead to a 35% increased risk for mortality [8]. Without biochemical tests, testosterone deficiency is difficult to diagnose because of its vague symptoms [9].

In this research, the ADAM questionnaire exhibits 90.12% sensitivity, 47.31% specificity, 45.34% positive predictive value, 90.80% negative predictive value, and 61.29% accuracy. The ADAM questionnaire showed adequate sensitivity but as it lacks adequate specificity we can conclude that it is not a good alternative for serum testosterone assays. This was consistent with a study conducted in Kenya which showed an 88.1% sensitivity and 44.7% specificity with 61.4% accuracy [10]. Chu et al. also found similar results in the Chinese population with 86% sensitivity and 40% specificity [11]. A similar study was conducted in the United States by Morley et al. reported 97% sensitivity and 30% specificity [12].

Ugwu et al. in their study discussed the reason for low specificity of the ADAM questionnaire. They mention that the ADAM questionnaire comprises three components; two of which (mood and energy) are susceptible to being affected by multiple factors and illnesses, other than androgen deficiency. This may be responsible for low specificity. They also determined the sensitivity and specificity for questions related to sexual functions. They found that specificity for the questionnaire for reduced libido is higher compared to the specificity of the ADAM questionnaire (75.5% vs. 44.7%). Based on this finding, Ugwu et al. recommended continuing using the ADAM questionnaire for screening, especially in low-income countries, as yes to the question related to loss of libido may be a good indicator for low testosterone levels [10].

As per our knowledge, this is the first study that has given the sensitivity, specificity and accuracy of the ADAM questionnaire for testosterone deficiency in Pakistan. However, there are certain limitations to this study. Total testosterone was used instead of bioavailable testosterone because of lack of resources. Also, all the samples were collected from a single center which narrows the results reserved to that population.

## Conclusions

Low specificity, positive predictive value, and accuracy were found in this study. However, sensitivity and negative predictive values were satisfactory. We suggest that doctors should screen patients with the ADAM questionnaire since questions related to sexual function on the ADAM questionnaire might be helpful in the diagnosis of patients with testosterone deficiency. Considering there are limited resources, a more robust history should be taken before sending blood samples to determine total testosterone level. Further clinical studies should be conducted to make a questionnaire to screen patients with testosterone deficiency.
